# Establishment of minigenomes for infectious bursal disease virus

**DOI:** 10.1186/s13567-024-01423-6

**Published:** 2024-12-18

**Authors:** Hui Yang, Mingrui Zhang, Sanying Wang, Daxin Peng, Luis Martinez-Sobrido, Chengjin Ye

**Affiliations:** 1 Disease Intervention and Prevention Program, Texas Biomedical Research Institute, San Antonio, TX USA; 2 College of Veterinary Medicine, Yangzhou University, Yangzhou, Jiangsu China; 3 Wenzhou Medical University, Affiliated Hospital 1, Wenzhou, Zhejiang China; 4 Zhejiang Key Laboratory of Geriatrics and Geriatrics Institute of Zhejiang Province, Zhejiang Hospital, Hangzhou, China

**Keywords:** IBDV, minigenomes, VP1, VP3, reporter gene, antivirals

## Abstract

Minigenomes (MGs) have greatly advanced research on the viral life cycle, including viral replication and transcription, virus‒host interactions, and the discovery of antivirals against RNA viruses. However, an MG for infectious bursal disease virus (IBDV) has not been well established. Here, we describe the development of IBDV MG, in which the entire coding sequences of viral genomic segments A and B are replaced with Renilla luciferase (Rluc) or enhanced green fluorescent protein (EGFP) reporter genes. Under the control of the RNA polymerase I promoter, the translation of IBDV MG is controlled by the viral proteins VP1 and VP3. Interestingly, IBDV B MG shows greater activity than does IBDV A MG. Moreover, the sense IBDV B MG was expressed at a higher level than the antisense IBDV B MG. In agreement with our previous findings, the translation of IBDV B MG controlled by VP1 and VP3 is independent of the cellular translation machinery components eukaryotic initiation factor (eIF)4E and eIF4G, but intact VP1 polymerase activity, VP3 dsRNA-binding activity, and the interaction between VP1 and VP3 are indispensable for both sense and antisense IBDV B MG activity. In addition, ribavirin, which inhibits IBDV replication, inhibits IBDV B MG activity in a dose-dependent manner. Collectively, the IBDV MG established in this study provides a powerful tool to investigate IBDV intracellular replication and transcription and virus‒host interactions and facilitates high-throughput screening for the identification of IBDV antivirals.

## Introduction

Infectious bursal disease (IBD) is an acute and highly contagious immunosuppressive disease of young (1–2-week-old) chickens and is a significant threat to the poultry industry [1–3]. IBD was first discovered in the United States and has quickly spread to major poultry breeding areas around the world [2, 3].

Infectious bursal disease virus (IBDV), which is a member of the *Birnaviridae* family, is the causative agent of IBD [4]. IBDV genome consists of two segments of double-stranded (ds) RNA: segments A and B [5]. Segment A contains two partially overlapping open reading frames (ORFs). The larger segment A ORF encodes a polyprotein that is cleaved to produce the mature viral proteins VP2, VP3 and VP4 [6]. VP2 and VP3 are the major viral structural proteins that form the viral capsid [7]. VP3 has been shown to be involved in antiapoptotic activities, preventing the activation of cellular dsRNA-dependent protein kinase R (PKR) [8]. VP3 has also been identified as an inhibitor of melanoma differentiation-associated protein 5 (MDA5) and prevents MDA5-mediated induction of interferon beta (IFNβ) by binding to IBDV genomic dsRNA [9]. The smaller segment A ORF encodes the nonstructural protein VP5 [10], which is not necessary for viral replication in cultured cells but plays an important role in viral pathogenesis in vivo [11, 12]. Segment B encodes VP1, a putative viral RNA-dependent RNA polymerase (RdRp) [13, 14]. In virions, IBDV VP1 is bound to both genome A and B segments [15, 16] and forms, together with VP3 and viral dsRNA, ribonucleoprotein (RNP) complexes, which are essential for viral genome replication and gene transcription [17–19].

There are two distinct serotypes of IBDV, serotypes I and II, with only serotype I being pathogenic in chicks [20]. According to differences in antigenicity and virulence, serotype I IBDVs are further classified into classical (cIBDV), antigenic (avIBDV), and very virulent (vvIBDV) strains [1]. Owing to differences in viral antigenicity, vaccination represents the best option to prevent cIBDV but does not work well with avIBDV and vvIBDV strains [21, 22]. With the discovery of new avIBDV and vvIBDV strains, IBDV control has become more challenging. Repurposing antiviral chemical compounds, which have been used for many other viruses, may be a way to control avIBDV and vvIBDV; however, a lack of assays compatible with high-throughput screening (HTS) has limited the ability to identify antivirals for the treatment of IBDV infection. Minigenome (MG) assays, which mimic intracellular viral genome replication and gene transcription, have been used for the identification and characterization of antivirals against different RNA viruses [23–25]. However, similar MG assays for IBDV have not yet been developed, limiting interrogation of compound libraries in HTS settings to identify antivirals for the treatment of IBDV infection.

Recently, we identified VP1 and VP3 as the minimal *trans*-acting factors for IBDV genome replication and transcription [26], which was subsequently confirmed by other groups [27]. The discovery of VP1 and VP3 as the minimum components for IBDV intracellular genome replication and gene transcription and the successful rescue of recombinant IBDV have paved the way for the development of IBDV MG. In this study, we establish IBDV MG approaches to assess viral replication and transcription under the control of the IBDV VP1 and VP3 proteins. Importantly, we demonstrate the feasibility of the use of these new IBDV MGs for the identification of antivirals that affect genome replication and gene transcription.

## Materials and methods

### Cells, viruses, and antibodies

HEK293T (ATCC; CRL-11268) and the chicken fibroblast cell line DF-1 (ATCC; CRL-12203) were routinely cultured at 37 °C with 5% CO_2_ in Dulbecco’s modified Eagle’s medium (DMEM) (Gibco, Carlsbad, CA, USA) supplemented with 10% foetal bovine serum (FBS). The IBDV strain (IBDV/WT) used in this study has been described in detail previously [28]. Mouse anti-VP1 and anti-VP3 polyclonal antibodies were produced as previously described [28]. Mouse anti-GAPDH and anti-FLAG monoclonal antibodies were obtained from BioBEST Biotechnology (Anhui, China). A rabbit anti-GFP monoclonal antibody was purchased from Abcam (Cambridge, MA, USA). Rabbit anti-eIF4E and anti-eIF4G monoclonal antibodies were obtained from Cell Signaling Technology (Danvers, MA, USA).

### Plasmid construction, site-directed mutagenesis and transfection

The plasmids p1-mA/p1-RmA and p1-mB/p1-RmB, which encompass the full IBDV genomic A and B sequences, served as the foundational vectors for creating the MG constructs used in this study [26]. Mutations within the IBDV UTR and VP1 were introduced via site-directed mutagenesis using Agilent technology (Santa Clara, CA, USA). All plasmids were confirmed by Sanger sequencing to ensure that no unwanted mutations were introduced. The sequences of primers used for constructing these plasmids are available upon request. The expression plasmids used for IBDV VP1, VP3, VP1/D402A, VP1/D416A, VP3/KRKK, and VP3/dC were described previously [26]. Additionally, a plasmid encoding firefly luciferase (Fluc) under a constitutively polymerase II promoter (pCAGGS-Fluc) was used. Transfections were carried out using transfection reagent (BioBEST) following the manufacturer’s guidelines, with plasmid quantities for each group maintained consistently through the use of the pCI-neo vector.

### Western blotting

Whole-cell lysates were prepared, separated by SDS‒PAGE, and analysed via western blotting as previously described [26, 29]. In brief, cell lysates were obtained by incubating the cells in lysis buffer (50 mM Tris-HCl pH 7.4, 150 mM NaCl, 1% Triton X-100, and 1% sodium deoxycholate) on ice for 30 min, followed by centrifugation at 12 000 × *g* for 5 min at 4 °C. Equal quantities of lysates were loaded onto 10% or 12% SDS‒PAGE gels and transferred to nitrocellulose membranes. The membranes were then blocked with 5% bovine serum albumin (BSA) in PBS with 0.1% Tween 20 (PBST) at room temperature for 1 h. Primary antibodies were applied and incubated overnight at 4 °C, followed by incubation with horseradish peroxidase-conjugated secondary antibodies for 1 h at 37 °C. GAPDH served as the loading control. Detection was performed using an enhanced chemiluminescence (ECL) reagent (BioBEST) and an Amersham or Bio-Rad imager. After visualization, the blots were scanned, and densitometric analysis was conducted with ImageJ software.

### MG assays

HEK293T cells were transfected with IBDV MGs expressing Renilla luciferase (Rluc) or enhanced green fluorescent protein (EGFP) reporter genes together with the indicated plasmids. The pCAGGS-Fluc plasmid was simultaneously transfected as an internal control and used to normalize the transfection and Rluc activities. After transfection, the cells were lysed with passive lysis buffer, and the Rluc and Fluc activities in the cell lysates were determined with the Dual-Luciferase Reporter Assay System (Promega) using a multiplate reader (BioTek). Alternatively, EGFP expression from IBDV MGs in transfected cells was examined under a fluorescence microscope (Zeiss).

### Drug treatment

Ribavirin was purchased from Beyotime Biotechnology (Jiangsu, China), dissolved in DMSO and used at final concentrations of 10 µM and 100 µM. HEK293T cells were treated with the indicated concentrations of ribavirin or the DMSO control at 24 h post-transfection with IBDV MG. Afterwards, the cells were harvested for MG activity at 72 h post-transfection. DF-1 cells infected with the DF-1 cell-adapted IBDV strain (MOI of 0.01) were treated with the same concentrations of ribavirin or the DMSO control, and cell culture supernatants were harvested for viral titrations using a standard plaque assay.

### Viral titrations

Viral titres were determined using plaque assays in DF-1 cells as described previously [26, 29]. Briefly, 10-fold serially diluted samples of cell culture supernatants were inoculated into DF-1 cell monolayers in 6-well plates. The cells were washed with PBS after viral inoculation at 37 °C for 1 h, after which the medium was replaced with 1% low-melting agarose. Finally, the cells were fixed with 4% formaldehyde and stained with 1% crystal violet at 96 h post-infection. Viral titres were determined by counting viral plaques.

### Alignment of the IBDV segment B UTR

The previously published nucleotide sequences of the IBV segment B UTR used in our analysis include A44 (MF083702.1), HK46 (AF092944.1) and the classical isolate Cu1 (AF362775.1). The nucleotide sequences of the IBDV segment B UTR were aligned using DNAMAN (Lynnon Biosoft, San Ramon, CA, USA).

### Statistical analysis

All the data are presented as the means ± standard deviation (SD) for each group and were analysed using SPSS 13.0 (IBM, Armonk, NY, USA). Student’s *t* test was used for comparisons between two groups. A *P* value of less than 0.05 (*P* < 0.05) was considered statistically significant.

## Results

### Development of IBDV MGs expressing Rluc or EGFP

**Figure 1:**
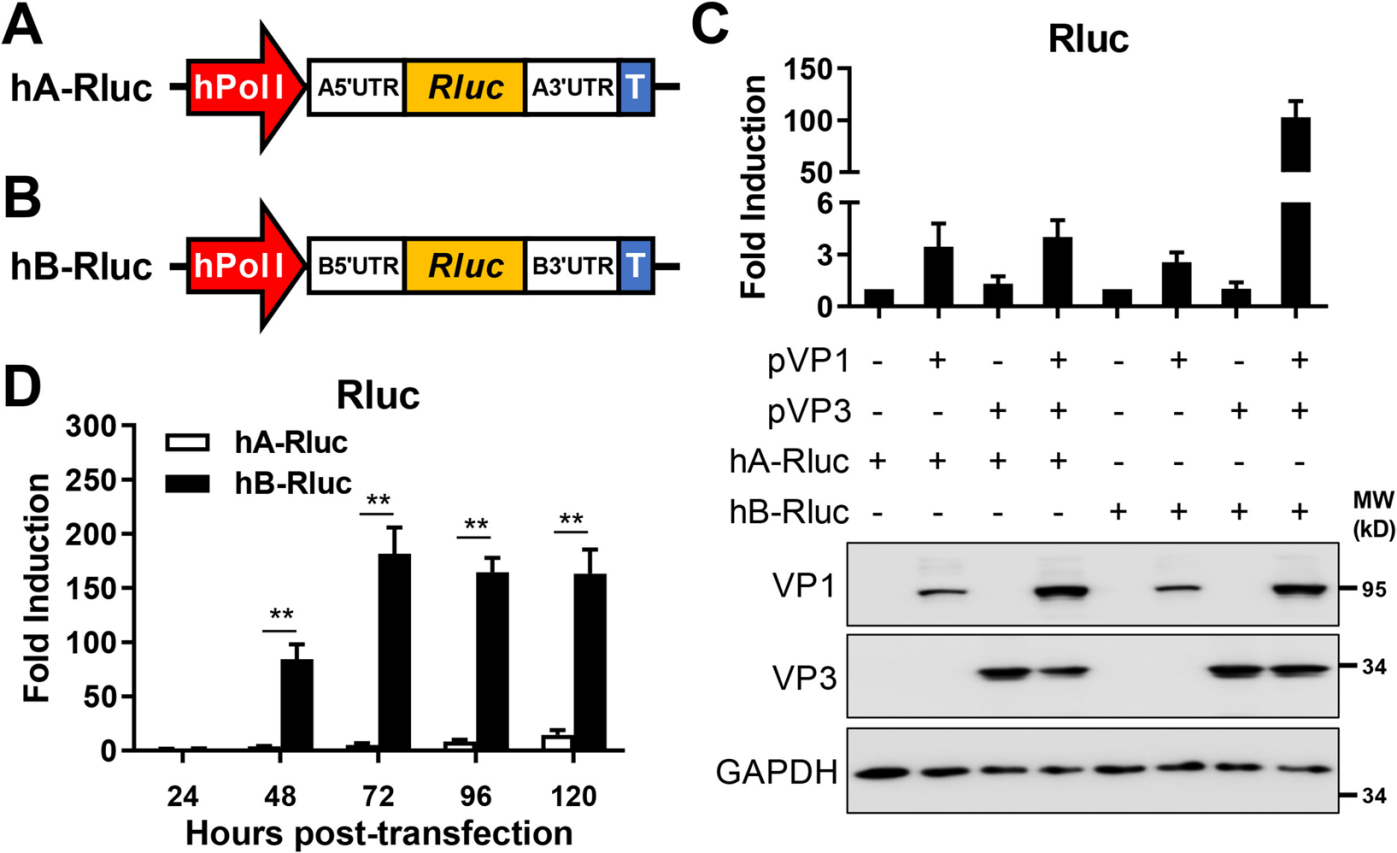
**IBDV minigenomes (MGs) expressing Renilla luciferase (Rluc).** **A** and **B** Schematic representation of the IBDV MG expressing Rluc on the basis of the A (**A**) or B (**B**) viral segment backbone. The human polymerase I promoter (Pol I, red) and the mouse polymerase I terminator (T, blue) represent the human polymerase I promoter and mouse terminator I, respectively. **C** HEK293T cells (6-well plate format, 10^6^ cells/well, triplicates) were transfected with Fluc (0.01 µg/well), hA-Rluc or hB-Rluc (2 µg/well), together with pVP1 (1 µg/well) or/and pVP3 (1 µg/well). The total amount of plasmids in each well was maintained at a consistent level by adding empty pCI-neo. At 48 h post-transfection, the cells were lysed using passive lysis buffer, and the Rluc and Fluc activities were determined using a dual luciferase detection kit and a multiplate reader. The expression of VP1, VP3, and GAPDH was determined by western blot analysis using the corresponding antibodies. The data are presented as the means ± SD and are representative of three independent experiments. **D** HEK293T cells (6-well plate format, 10^6^ cells/well, triplicates) were transfected with Fluc (0.01 µg/well), hA-Rluc or hB-Rluc (2 µg/well), together with the pVP1 (1 µg/well) or/and pVP3 (1 µg/well) plasmids. The total amount of plasmids in each well was maintained at a consistent level by adding empty pCI-neo. At the indicated times after transfection, the cells were lysed in lysis buffer, and the Rluc and Fluc activities were determined using a multiplate reader. The data are *p* < 0.01.

The human RNA polymerase I promoter (hpol-I) has been shown to produce viral genome copies that are efficiently recognized by viral polymerase complexes to initiate viral replication and transcription [26]. To develop a reliable MG for IBDV, we generated two hpol-I-controlled plasmids based on either IBDV segment A (hA-Rluc) or B (hB-Rluc) by substituting the entire viral coding sequence for Rluc (Figures 1A and B). To test the ability of IBDV VP1 and VP3 to efficiently replicate and transcribe these MGs, hA-Rluc or hB-Rluc plasmids were cotransfected with the expression plasmids pVP1 and/or pVP3. A plasmid encoding Fluc under a polymerase II promoter was included as an internal control and used to normalize the transfection and Rluc activities. Rluc showed approximately 100-fold activity in cells co-transfected with pVP1, pVP3, and hB-Rluc at 48 h post-transfection (Figure 1C). Importantly, the expression of IBDV VP1 and VP3 was comparable in both MG assays (Figure 1C). Moreover, hB-Rluc activity peaked at 72 h post-transfection and lasted for 120 h post-transfection without significant reduction (Figure 1D). hA-Rluc MG activity was also activated but was lower (~15-fold) than that of hB-Rluc (~160-fold) at 120 h post-transfection (Figure 1D).

**Figure 2:**
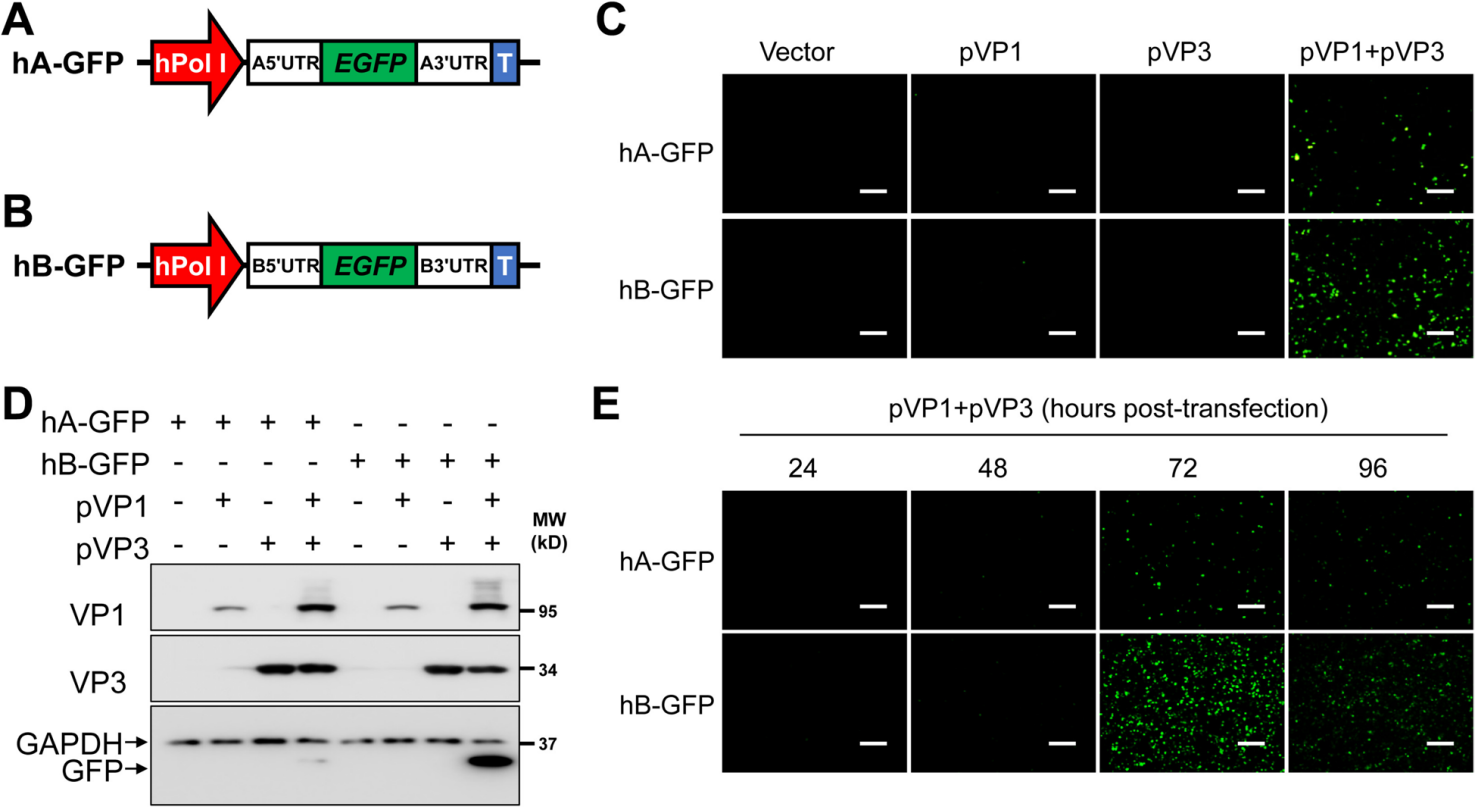
**IBDV MG expressing enhanced green fluorescence protein (EGFP).** **A** and **B** Schematic representation of IBDV MG expressing EGFP on the basis of the A (**A**) or B (**B**) viral segment backbones. hPol I (red) and T (blue) represent the human Pol I promoter and mouse terminator I, respectively. **C** and **D** HEK293T cells (6-well plate format, 10^6^ cells/well, triplicates) were transfected with hA-GFP or hB-GFP (2 µg/well), together with pVP1 (1 µg/well) or/and pVP3 (1 µg/well). At 72 h post-transfection, the expression of EGFP was determined using an inverted fluorescence microscope (**C**) or by western blot analysis of whole-cell lysates (**D**). **E** HEK293T cells (6-well plate format, 10^6^ cells/well, triplicates) were cotransfected with hA-GFP or hB-GFP (2 µg/well), together with pVP1 (1 µg/well) and pVP3 (1 µg/well) plasmids. The expression of EGFP was determined using an inverted fluorescence microscope at the indicated times post-transfection. Representative images are shown. Scale bar, 200 μm. Magnification, ×10.

To directly visualize MG activity, we generated similar MG plasmids expressing EGFP (hA-EGFP and hB-EGFP) using the same strategy (Figures 2A and B). The activation of the IBDV EGFP-expressing MG also relied on VP1 and VP3 expression. The segment B-based MG expressed higher levels of EGFP than hA-EGFP did (Figures 2C and E) in the presence of comparable levels of IBDV VP1 and VP3 expression (Figure 2D), which is consistent with the Rluc-expressing MG (Figure 1). These data demonstrate that both the IBDV MG expressing Rluc or EGFP under the control of hpol-I were dependent on IBDV VP1 and VP3 expression and that the IBDV segment B-derived MG expressed higher levels of Rluc or EGFP reporter genes than did the IBDV segment A-derived MG.

### Generation and reporter activity of antisense IBDV B MG

**Figure 3:**
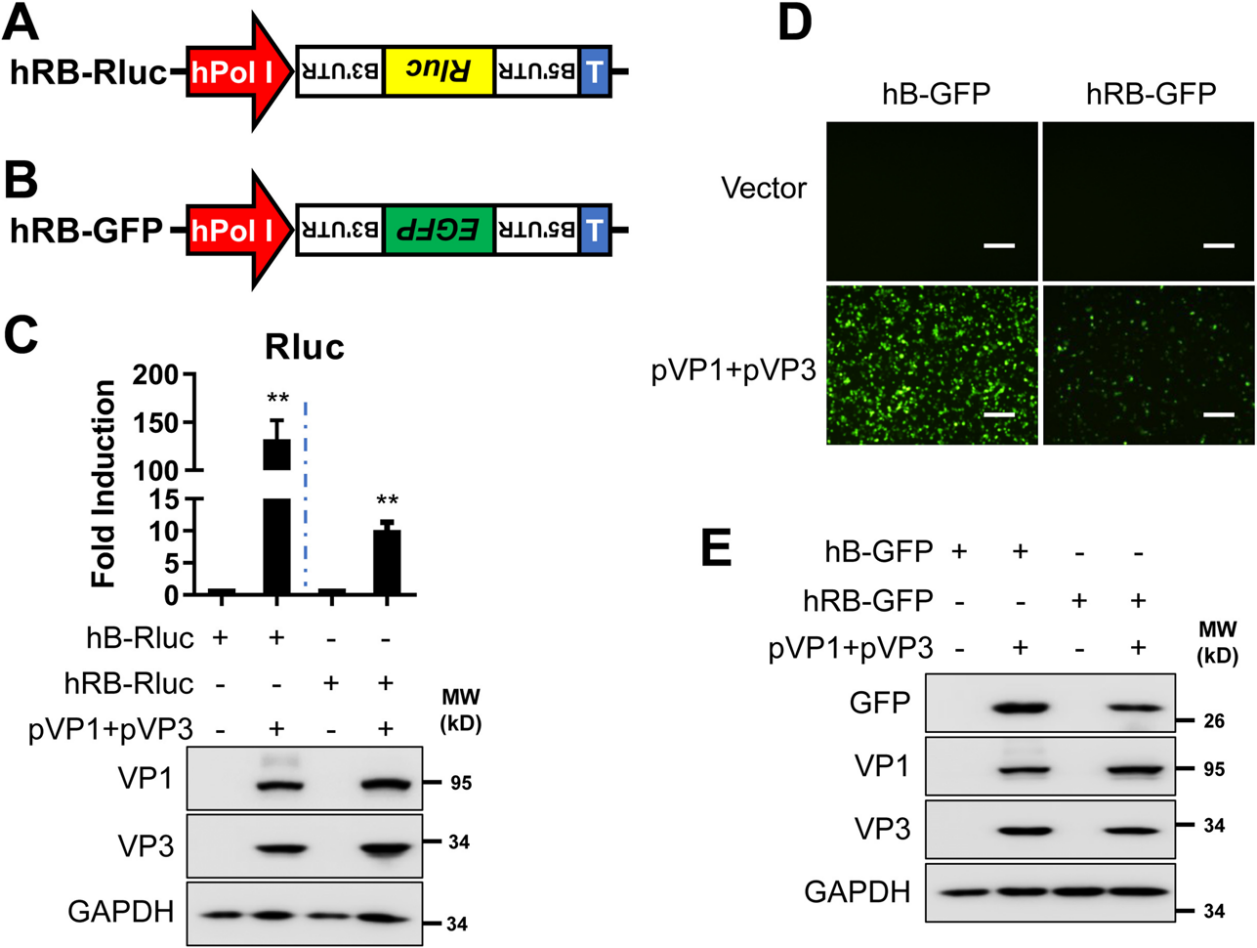
**Comparison of the activities of the sense and antisense IBDV MG.** **A** and **B** Schematic representations of IBDV antisense hRB-Rluc (**A**) and hRB-GFP (**B**) reporter plasmids for the MG based on the B segment backbone. hPol I (red) and T (blue) represent the human Pol I promoter and mouse terminator I. **C** HEK293T cells (6-well plate format, 10^6^ cells/well, triplicate) were transfected with Fluc (0.01 µg/well), hB-Rluc or hRB-Rluc (2 µg/well), together with pVP1 and pVP3 (1 µg/well each). At 72 h post-transfection, the cells were lysed in passive buffer, and the Rluc and Fluc activities were measured using a multiplate reader. The expression of VP1, VP3, and GAPDH was analysed by western blotting. The data are presented as the means ± SD and are representative of three independent experiments. **, *p* < 0.01. **D** and **E** HEK293T cells (6-well plate format, 10^6^ cells/well, triplicate) were cotransfected with hB-GFP or hRB-GFP plasmids alone or together with pVP1 and pVP3 plasmids. Fluorescence microscopy (**D**) and western blotting (**E**) were performed to determine the expression of EGFP and viral proteins using specific antibodies. GAPDH was used as a loading control. Scale bar, 200 μm. Magnification, ×10.

To examine whether the IBDV B MG with antisense polarity is more efficient than our previous construct in the sense orientation, we constructed antisense IBDV B MG expressing either Rluc or EGFP (Figures 3A and B). In cells transfected with hRB-Rluc MG, Rluc activity was ~ 10-fold greater than that of hB-Rluc MG, which was ~ 150-fold greater at 72 h post-transfection (Figure 3C). Similarly, EGFP expression in the hRB-EGFP MG was lower than that in the hB-EGFP MG (Figure 3D), which was also confirmed by western blot analysis of EGFP expression in the transfected cell lysates (Figure 3E). These data indicate that the IBDV B MG in the sense orientation results in more robust Rluc or EGFP reporter expression than does the antisense IBDV B MG and is dependent on the expression of the IBDV VP1 and VP3 proteins.

### Activation of IBDV MG depends on intact viral RNP activity

**Figure 4:**
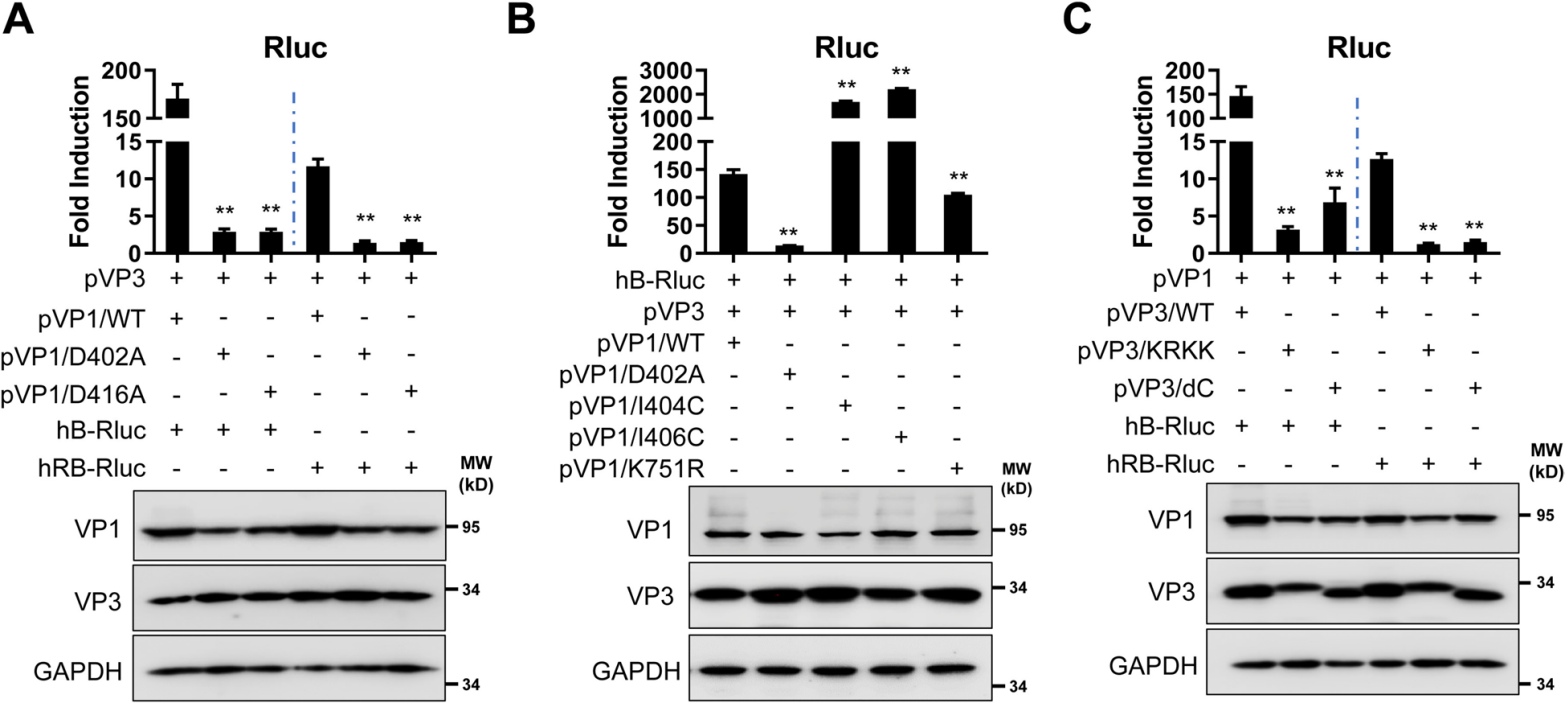
**Effects of VP1 and VP3 on IBDV MG activity.** **A** HEK293T cells (6-well plate format, 10^6^ cells/well, triplicate) were transfected with plasmids expressing Fluc (0.01 µg/well), VP1 (1 µg/well) wild type (WT), D402A or D416A, and pVP3 plasmid (1 µg/well) together with hB-Rluc or hRB-Rluc plasmids (2 µg/well). Luciferase assays were performed at 72 h after transfection. The expression of VP1 and VP3 was determined by western blotting. GAPDH was used as a loading control. **B** HEK293T cells (6-well plate format, 10^6^ cells/well, triplicates) were transfected with plasmids expressing Fluc (0.01 µg/well), VP1 (1 µg/well) WT or the indicated mutants and with the pVP3 plasmid (1 µg/well), together with the hB-Rluc plasmid (2 µg/well). Luciferase assays were performed at 72 h after transfection. The expression of VP1 and VP3 was determined by western blotting. GAPDH was used as a loading control. **C** HEK293T cells (6-well plate format, 10^6^ cells/well, triplicates) were transfected with plasmids expressing Fluc (0.01 µg/well), VP3 (1 µg/well) WT or mutants and the pVP1 plasmid (1 µg/well) together with hB-Rluc or hRB-Rluc (2 µg/well). Luciferase assays were performed at 72 h after transfection. The expression of VP1 and VP3 was further determined by western blotting. GAPDH was used as a loading control. The data are presented as the means ± SD and are representative of three independent experiments. **, *p* < 0.01.

VP3 interacts with both IBDV genomic dsRNA and VP1 to form viral RNP complexes, so we examined whether the RdRp activity of VP1, the dsRNA binding activity of VP3, and the association between VP1 and VP3 are required for the activation of the IBDV B MG. The RdRp-deficient VP1 mutants D402A and D416A failed to activate the sense or antisense IBDV B MGs, as determined by Rluc expression (Figure 4A). We next tested modifications of IBDV VP1, including ubiquitination (K751R) and SUMOylation (I404C and I406C) [30, 31]. These mutations in IBDV VP1 were shown to significantly decrease viral replication. Surprisingly, none of the IBDV VP1 mutants containing I404C or I406C failed to replicate and transcribe the IBDV MG. Only the VP1 mutants D402A and K751R showed defects or weak MG activity, respectively (Figure 4B). Notably, MG activity with the I404C and I406C VP1 mutants was approximately 10–15-fold greater than that with the IBDV VP1 WT (Figure 4B). As expected, mutants affecting the dsRNA binding activity of IBDV VP3 (KRKK and VP3dC) affected MG activity with both the sense and antisense IBDV B MG, as shown by Rluc expression (Figure 4C). These results demonstrate the feasibility of using the IBDV MG assay to assess the contribution of VP1 or VP3 mutations to viral genome replication or gene transcription.

### Effect of N- or C-terminal FLAG tags in IBDV VP1 and VP3 on MG activity

**Figure 5:**
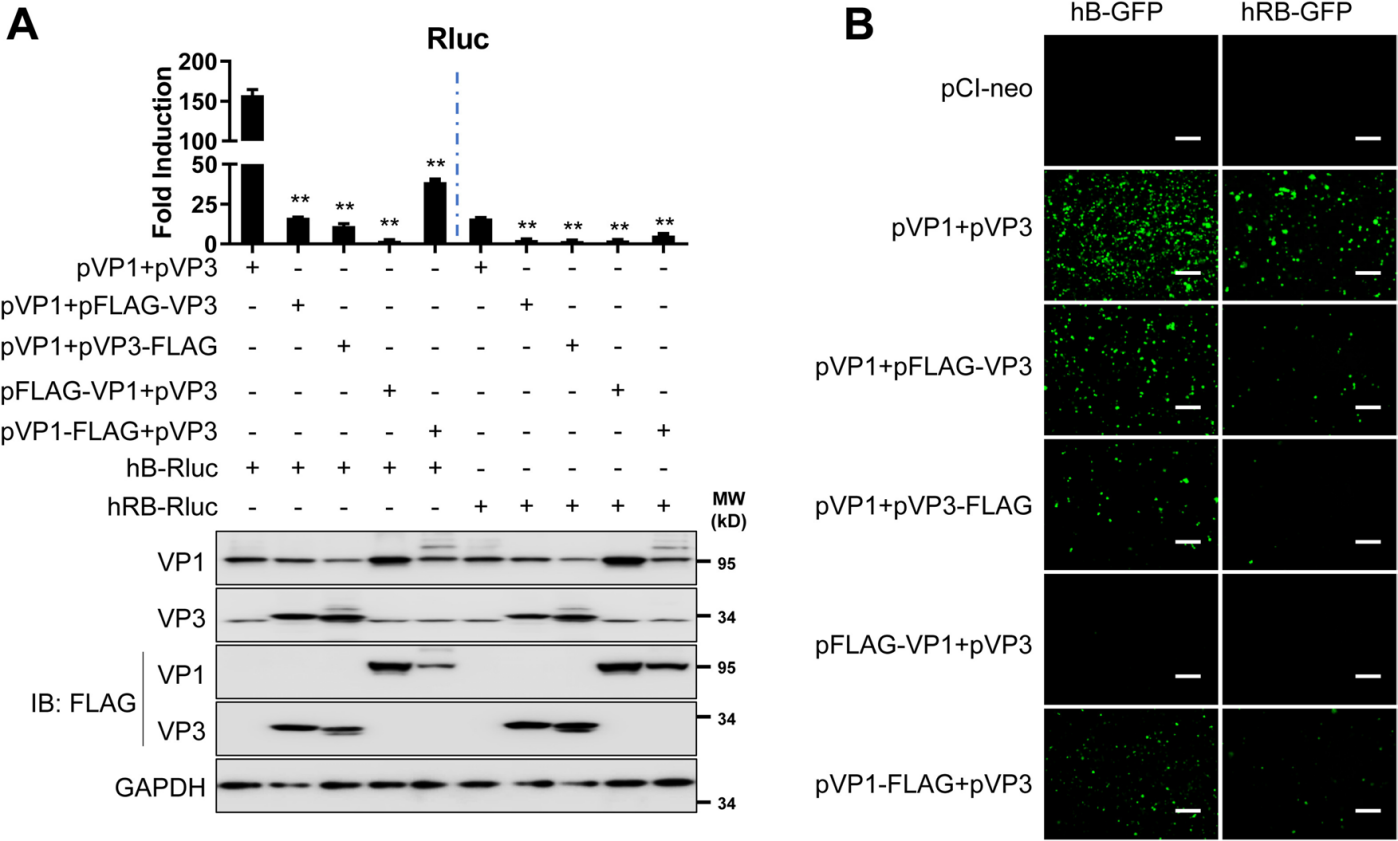
**Effect of a FLAG tag in the IBDV RNP components on MG activity.** **A** HEK293T cells (6-well plate format, 10^6^ cells/well, triplicate) were co-transfected with the indicated plasmids. Luciferase assays were performed at 72 h after transfection. The expression of VP1 and VP3 was determined by western blotting with the indicated antibodies. GAPDH expression was used as a loading control. The data are presented as the means ± SD and are representative of three independent experiments. **, *p* < 0.01. **B** HEK293T cells (6-well plate format, 10^6^ cells/well, triplicates) were co-transfected with the indicated plasmids, and EGFP expression was determined at 72 h post-transfection using an inverted fluorescence microscope. Scale bar, 200 μm. Magnification, ×10.

Recently, the FLAG tag, which is composed of 8 amino acids (DYKDDDDK), was shown to reduce IBDV RNP activity when it is fused to the N- or C-terminus of VP1 or VP3 [28]. To assess the contribution of the FLAG tag to IBDV VP1 and VP3 MG activity, we constructed N- and C-terminal FLAG-tagged versions of IBDV VP1 and VP3 that were tested in the MG assay. Rluc activity was significantly decreased in cells transfected with IBDV VP1 or VP3 fused to the FLAG tag on the N- or C-terminus, even when the N-terminal or C-terminal FLAG tag did not affect VP1 or VP3 protein expression (Figure 5A). Similar results were observed when the IBDV B MG expressing EGFP was used (Figure 5B). These data demonstrate that a FLAG tag on the N- or C-terminal domain of IBDV VP1 or VP3 affects viral genome replication and gene transcription, indicating that an intact IBDV RNP is required for efficient viral MG activity.

### IBDV B MG activation is independent of eIF4E and eIF4G

**Figure 6:**
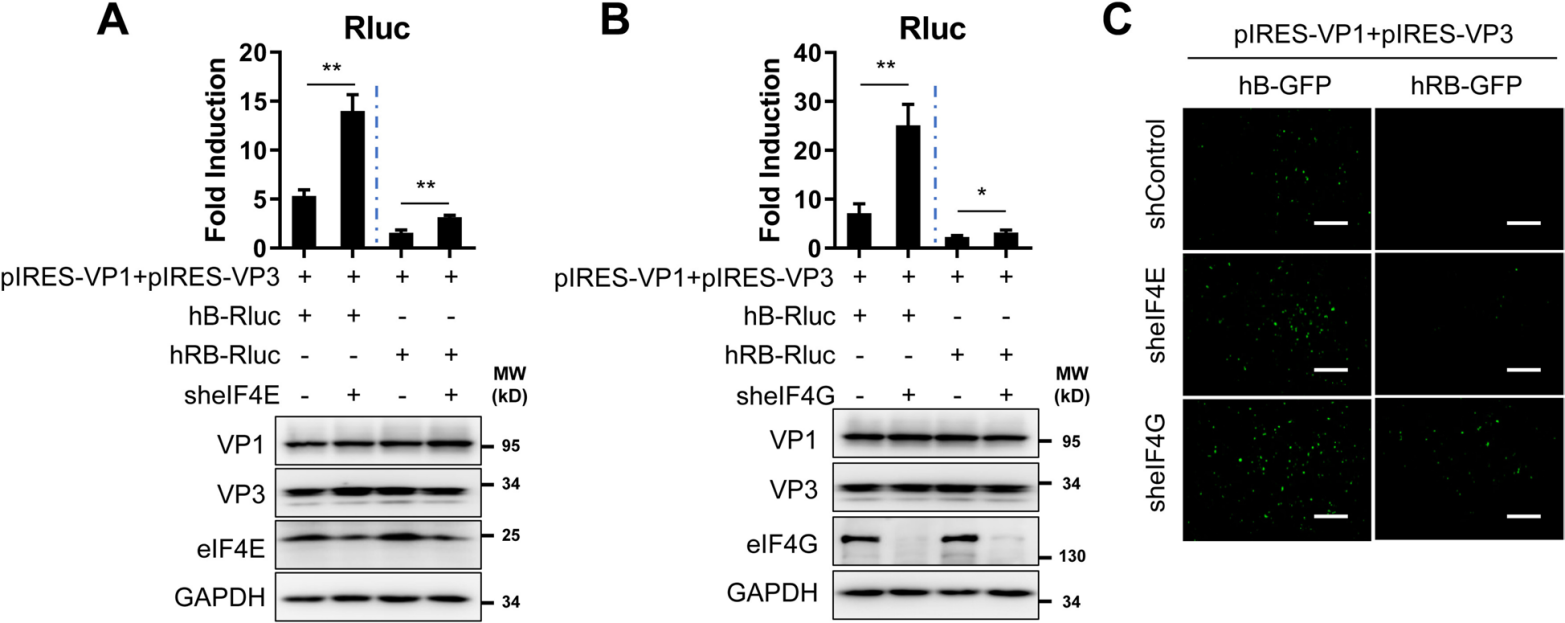
**IBDV MG activity is independent of eIF4E and eIF4G.** **A** and **B** HEK293T cells (6-well plate format, 10^6^ cells/well, triplicate) were transfected with shRNA plasmids for 24 h. After shRNA plasmid transfection, the cells were co-transfected with the indicated plasmids. Luciferase assays were performed at 72 h after transfection. The expression of VP1 and VP3 was determined by western blotting with the indicated antibodies. GAPDH was used as a loading control. The data are presented as the means ± SD and are representative of three independent experiments. **, *p* < 0.01 and *, *p* < 0.05. **C** HEK293T cells (6-well plate format, 10^6^ cells/well, triplicate) were transfected with the shRNA plasmids and co-transfected for 24 h with the indicated plasmids. At 72 h, the expression of EGFP was determined by fluorescence microscopy. Scale bar, 200 μm. Magnification, ×10.

The eukaryotic initiation factors eIF4E and eIF4G are key components for mediating the translation of cell-capped mRNAs [32, 33]. EIF4E recognizes and binds the m7G cap structure at the 5′ terminus of cellular mRNA [34]. EIF4G serves as a scaffolding protein to recruit other translation initiation factors, such as eIF4A, eIF4B, and poly(A)-binding protein, by interacting with eIF4E [35, 36]. To test whether eIF4E or eIF4G are required for IBDV B MG activity, RNA interference was used to downregulate the expression of eIF4E or eIF4G. Downregulation of eIF4E resulted in increased (~ 2–3-fold) Rluc activity in both IBDV hB-Rluc and hRB-Rluc MG (Figure 6A). Similarly, downregulation of eIF4G resulted in an ~ 3-fold increase in IBDV B MG Rluc activity (Figure 6B). These results were confirmed with the EGFP-expressing IBDV B MG (Figure 6C). These data indicate that VP1/VP3-mediated IBDV B MG activation is independent of eIF4E and eIF4G, which are the most important components for cellular mRNA translation, suggesting the existence of a specific mechanism utilized by IBDV to mediate viral protein translation.

### Use of IBDV B MG for the identification of antivirals

**Figure 7:**
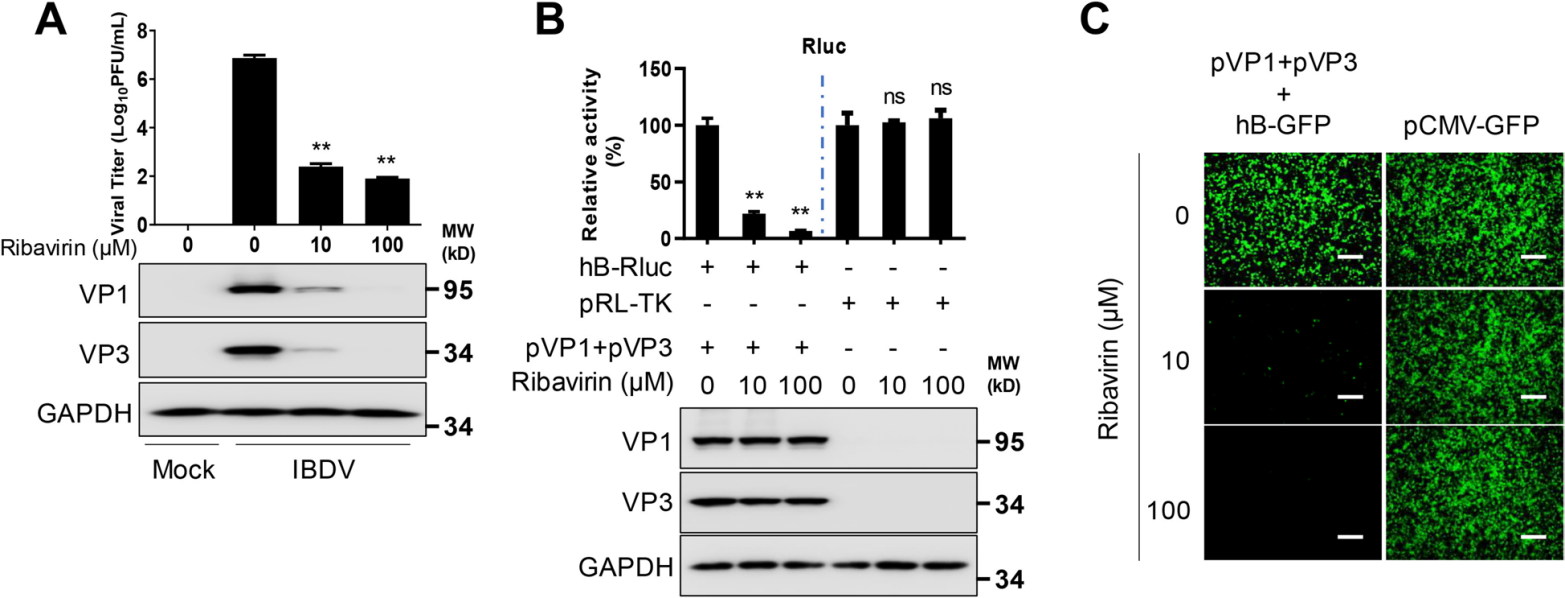
**Using IBDV MG for the identification of antivirals.** **A** Viral titres in DF-1 cells (6-well plate format, 10^6^ cells/well, triplicate) infected (MOI = 0.01) with IBDV and treated with ribavirin. Viral proteins were detected by western blotting. GAPDH was used as a loading control. **B** HEK293T cells (6-well plate format, 10^6^ cells/well, triplicates) were transfected with hB-Rluc (2 µg/well), pVP1 (1 µg/well), pVP3 (1 µg/well), or Fluc (0.01 µg/well). Alternatively, the cells were transfected with pRL-TK (0.1 µg/well). At 24 h post-transfection, media containing the indicated concentrations of ribavirin were added to each of the wells. At 72 h post-transfection, the cells were lysed with passive lysis buffer, and Rluc activity was determined. Viral proteins were detected by western blotting. GAPDH was used as a loading control. **C** HEK293T cells (6-well plate format, 10^6^ cells/well, triplicate) were transfected with hB-GFP (2 µg/well), pVP1 (1 µg/well) or pVP3 (1 µg/well). Cells transfected with pCI-EGFP were included as controls. At 24 h post-transfection, media containing the indicated concentrations of ribavirin were added to each of the wells. At 72 h post-transfection, the expression of EGFP was determined by an inverted fluorescence microscope. Representative images are shown. Scale bar, 200 μm. Magnification, ×10. The data are presented as the means ± SD and are representative of three independent experiments. **, *p* < 0.01.

Since IBDV B MG activity depends on the ability of the intact viral RNP to replicate and transcribe the viral genome, we tested the possibility of using IBDV B MG to identify antivirals against IBDV. To that end, we first tested the broad-spectrum antiviral agent ribavirin (virazole, 1-b-D-ribofuranosyl-1,2,4-triazole-3-carboxamide), which has been shown to inhibit IBDV intracellular replication (Figure 7A). Ribavirin treatment inhibited IBDV B MG activity in a dose-dependent manner (Figure 7B), and this inhibition was not due to a direct effect on Rluc because it did not affect Rluc expression from a constitutively expressing pRL-TK plasmid (Figure 7B). Similar dose-dependent ribavirin inhibition was observed in cells transfected with the IBDV B MG expressing EGFP (Figure 7C). These results demonstrate the feasibility of using IBDV MG to identify compounds affecting viral replication and transcription via Rluc or EGFP expression, which could be implemented for HTS to identify new compounds for treating IBDV infection.

### Effect of IBDV B 5ʹ and 3ʹ UTR on MG activity

**Figure 8:**
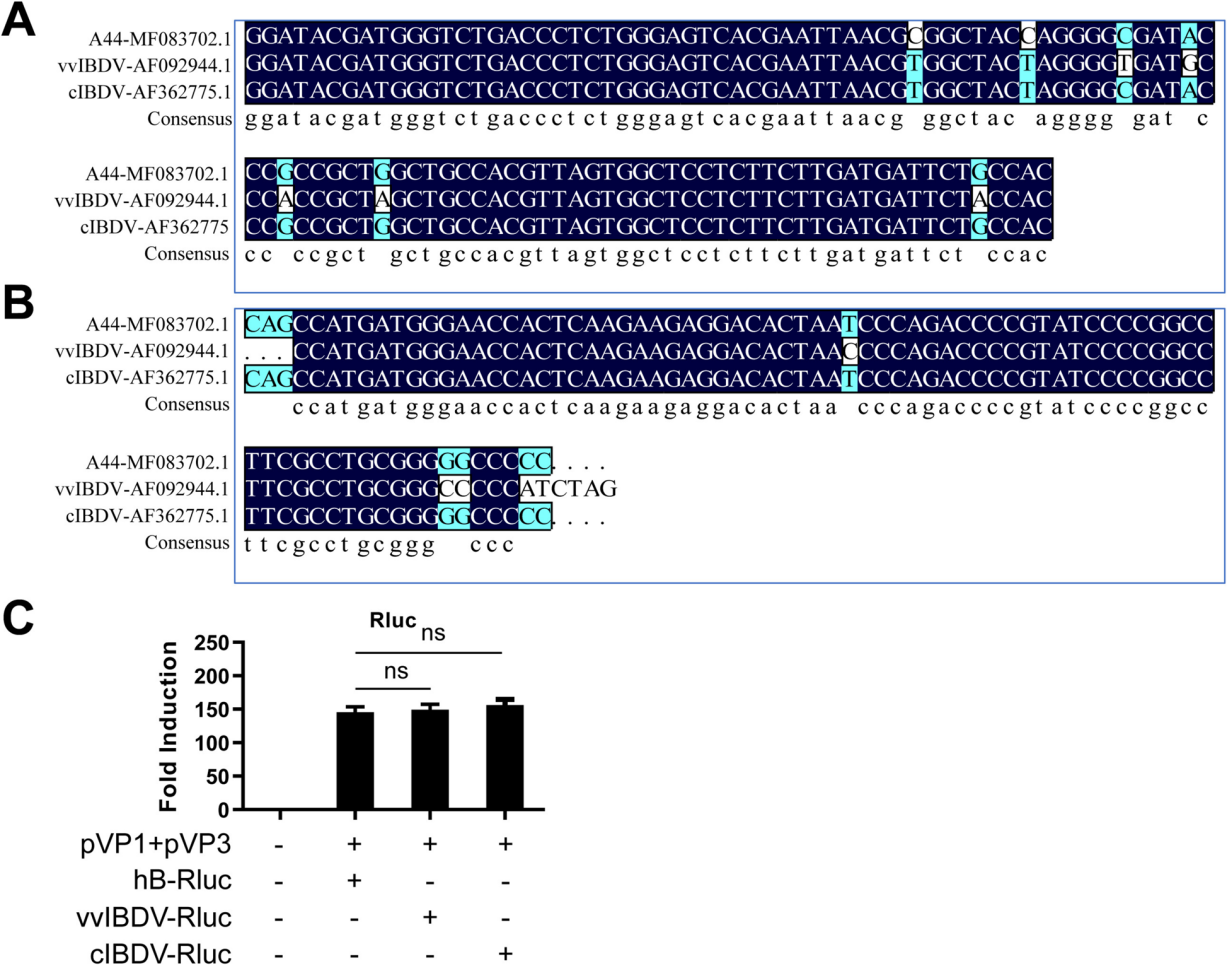
**Sequence differences in the IBDV B segment UTR do not affect MG activity.** **A** Nucleotide alignment of the IBV segment B 5ʹ UTR. **B** Nucleotide alignment of the IBV segment B 3ʹ UTR. **C** HEK293T cells (6-well plate format, 10^6^ cells/well, triplicate) were transfected with the indicated WT or modified hB-Rluc (2 µg/well), Fluc (0.01 µg/well), pVP1 (1 µg/well) or pVP3 (1 µg/well). The total amount of plasmids in each well was maintained at a consistent level by adding empty pCI-neo. At 72 h post-transfection, the cells were lysed in passive lysis buffer, and Rluc activity was determined via a multiplate reader. The data are presented as the means ± SD and are representative of three independent experiments.

Since different IBDV strains possess nucleotide polymorphisms in the segment B UTR, we sought to examine whether those different nucleotides in the 5ʹ and 3ʹ segment B IBDV UTR affect viral genome replication or gene transcription. Three representative B segment UTR sequences for each IBDV variant were aligned (Figures 8A and B). Nucleotide differences between these viral strains were introduced into the IBDV B MG plasmids by site-directed mutagenesis, and the resulting MGs were compared by assessing Rluc activity. In all cases, Rluc expression was induced by ~ 140–170-fold in cells co-transfected with pVP1 and pVP3, independent of the segment B 5ʹ and 3ʹ UTR (Figure 8C). These results demonstrate that polymorphisms in the IBDV segment B 5ʹ and 3ʹ UTR do not significantly affect viral genome replication or gene transcription, suggesting that differences in the segment B UTR may not contribute to differences in the pathogenicity of different IBDV strains. Importantly, these results support the possibility of identifying antivirals that target the segment B 5’ or 3’ UTR as a way to treat different IBDV strains.

## Discussion

IBD is one of the most important poultry immunosuppressive diseases and may induce temporary or permanent destruction of the lymphoid tissue in the bursa [1, 2]. Vaccination is the main means of preventing IBD, but the emergence of avIBDV and vvIBDV has resulted in reduced vaccine protection efficiency. Since no specific drugs have been approved for the treatment of avIBDV and vvIBDV infection, IBDV has spread worldwide and has presented significant challenges for the prevention and control of IBD in the poultry industry [3, 37]. IBDV genome replication and gene transcription are complicated biological processes involving viral RdRp complexes and cellular host factors. To date, no drug that targets IBDV genomic RNA replication or gene transcription has been approved. The identification and implementation of anti-IBDV drugs has been hampered by the lack of effective assays suitable for HTS. To that end, our cell-based MG assay represents an excellent option for recapitulating IBDV genome replication and transcription, with the additional advantage of having fluorescent (EGFP) or luciferase (Rluc) reporter genes that can be easily used to identify and characterize IBDV antivirals. Our studies using the broad-spectrum antiviral drug ribavirin with the IBDV MG validated the use of this approach for the identification of novel anti-IBDV drugs that target viral replication and transcription.

MGs are useful tools for investigating virus replication and transcription, virus‒host interactions, and antiviral screening. IBDV VP1 and VP3 have been shown to be required for IBDV genome replication and transcription, as well as for the recovery of infectious viruses via human polymerase I (hpol-I) promoter-based plasmids [26]. In this study, IBDV MGs were developed for use in human HEK293T cells because of their high transfection efficiency, the possibility of discovering the cellular factors involved in IBDV replication and transcription, and the ability to identify antivirals via a relevant human cell line. On the basis of our previous studies, we speculate that the IBDV MG will not work in avian cells because of the species specificity of polymerase I promoters. Thus, for further implementation of a similar IBDV MG to be functional in avian cells, similar MG constructs under an avian polymerase I promoter are needed.

MG systems for RNA viruses have been developed on the basis of the use of different promoters, including the T7, pol-I, or pol-II promoter. Our study presents the first pol-I-driven MG system for IBDV. By using these IBDV MG systems, we confirmed that the IBDV VP1 and VP3 proteins, as well as the viral UTR, are the minimal *trans*- and *cis-*factors required for viral genome replication and gene transcription. We further validated this finding by using both IBDV VP1 and VP3 mutant proteins. Interestingly, the activity of the IBDV MG expressing Rluc or EGFP reporter genes to identify IBDV segment B was better than that of IBDV segment A, which is contrary to the current knowledge that segment A is expressed more than segment B during IBDV infection. Similarly, we found that the MG activity of IBDV was greater than that of IBDV antisense MG activity. Surprisingly, the expression of reporter genes from the IBDV MG was independent of the host cellular factors eIF4E and eIF4G. We speculate that sequences in the IBDV 5ʹ and 3ʹ UTR might be involved in IBDV regulation of viral genome replication or gene transcription. Our studies suggest that nucleotide differences in the 5ʹ and 3ʹ UTR of 3 different IBDV strains do not play a significant role in reporter gene expression with our MG plasmids. However, other nucleotide residues in the 5ʹ and 3ʹ UTR may play important roles in viral replication and transcription. Likewise, it is possible that coding sequences are required for segment A and B expression during viral infection. To test this hypothesis, our IBDV A and B MG plasmids represent a unique opportunity to investigate whether noncoding or coding sequences are important for the expression of IBDV segments A and B.

In summary, we have established and validated a simple, convenient, and reliable IBDV MG system that can be used to answer important questions about the biology of IBDV, including viral replication, transcription, and virus‒host interactions, as well as the identification and characterization of novel drugs for the treatment of IBDV infection.

## Data Availability

All the data generated or analysed during this study are included in this published article.
